# Sleep Quality and Its Association with Health-Related Clinical Outcomes and Biomarkers Among Rural Northern Thai Older Adults: A Cross-Sectional Study

**DOI:** 10.3390/healthcare14152364

**Published:** 2026-08-03

**Authors:** Manuchet Manotham, Parichat Ong-Artborirak, Nestor Asiamah, Wannita Sakulwattana, Sriwan Chutithamniti, Wichida Larsomsri, Surangkana Chairinkam, Katekaew Seangpraw, Phatcharee Pimalram, Prapaporn Tulwattanakul

**Affiliations:** 1 School of Public Health, University of Phayao, Phayao 56000, Thailand; manuchet.ma@up.ac.th (M.M.); wannita.sa@up.ac.th (W.S.); wichida.la@up.ac.th (W.L.); surangkana.ch@up.ac.th (S.C.); eungkaew@gmail.com (K.S.); phatcharee.pi@up.ac.th (P.P.); 2 Department of Research and Medical Innovation, Faculty of Medicine Vajira Hospital, Navamindradhiraj University, Bangkok 10300, Thailand; ong.artborirak@gmail.com; 3 School of Health and Social Care, University of Essex, Colchester CO4 3SQ, UK; nestor.asiamah@myport.ac.uk; 4 Ban Pang Kha Health Promotion Hospital, Phayao 56140, Thailand; sriwan.chutitham@gmail.com

**Keywords:** sleep quality, biomarkers, older adults, non-communicable diseases, Thailand

## Abstract

Background/Objectives: Sleep disorders are common among older adults and have been increasingly linked to metabolic abnormalities and non-communicable diseases (NCDs). However, evidence on the relationship between sleep quality and health-related clinical outcomes and biomarkers in rural Northern Thailand remains limited. This study aimed to assess the association between sleep quality and health-related clinical outcomes and biomarkers among older adults in rural Northern Thailand. Methods: A cross-sectional study was conducted among 424 older adults in Phayao Province, rural Northern Thailand. Quota sampling was employed to ensure equal representation of participants with and without NCDs (*n* = 212 per group). Sleep quality was assessed using the Pittsburgh Sleep Quality Index (PSQI). Physical health status was evaluated through measurements of blood pressure (BP), including systolic blood pressure (SBP) and diastolic blood pressure (DBP), and fasting blood sugar (FBS). Results: The participants’ mean PSQI score was 8.03 ± 4.86, and 29.7% had poor sleep quality (PSQI > 5). Hypertension (35.4%) and diabetes mellitus (17.5%) were the most common underlying diseases. The mean fasting blood sugar level was 116.33 ± 30.84 mg/dL, with 21.0% of participants presenting abnormal levels (≥126 mg/dL). Multivariable binary logistic regression demonstrated that each one-point increase in the overall PSQI score was significantly associated with higher odds of having an underlying disease (adjusted odds ratio [AOR] = 2.37; 95% confidence interval [CI]: 2.00–2.81), hypertension (AOR = 1.51; 95% CI: 1.37–1.66), diabetes mellitus (AOR = 1.42; 95% CI: 1.28–1.58), other non-communicable diseases (AOR = 1.94; 95% CI: 1.64–2.29), abnormal fasting blood sugar (AOR = 1.47; 95% CI: 1.33–1.63), elevated systolic blood pressure (AOR = 1.39; 95% CI: 1.28–1.51), and elevated diastolic blood pressure (AOR = 1.30; 95% CI: 1.19–1.43). Subjective sleep quality, sleep duration, and habitual sleep efficiency were also significantly associated with multiple health-related clinical outcomes and biomarkers. Although sparse data and quasi-complete separation may have influenced the precision of some regression estimates, the overall pattern of associations remained consistent and should be interpreted with appropriate caution. Conclusions: Higher overall PSQI scores were significantly associated with adverse health-related clinical outcomes and biomarkers among older adults in rural Northern Thailand. These findings highlight the clinical importance of routinely assessing sleep quality in older adults, particularly those with chronic conditions or increased cardiometabolic risk. Because of the cross-sectional design and the statistical uncertainty associated with sparse data in some regression models, the findings should be interpreted as evidence of associations rather than causal relationships. The observed associations are consistent with the likely bidirectional relationship between sleep disturbances and chronic diseases. Integrating sleep quality screening into routine health assessments, alongside comprehensive chronic disease management, may facilitate the early identification of older adults at increased cardiometabolic risk and support evidence-based health promotion strategies.

## 1. Introduction

The global population of older adults is increasing rapidly, from 205 million individuals aged 60 years and older at present to a projected 2 billion by 2050 [1]. Insomnia is a prevalent condition among older adults that remains underrecognized, underdiagnosed, and inadequately treated [2]. It has been linked to adverse health outcomes [3,4] and is associated with an increased risk of mental health disorders, particularly depression and anxiety [5,6]. According to the World Health Organization, insomnia is defined in the Composite International Diagnostic Interview version 3 as the occurrence of at least one of the following nighttime difficulties: difficulty initiating sleep, difficulty maintaining sleep, early morning awakening, or non-restorative sleep, nearly every night for at least two weeks [7]. Epidemiological evidence suggests that 10–30% of the global population experiences chronic insomnia, with some estimates as high as 50–60% [8]. Older adults are particularly affected, with prevalence rates ranging from 30 to 48% depending on the population and study period [1]. Cross-national data further report severe to very severe sleep disturbances in up to 16.6% of older adults [9]. Consistent with global trends, national data from Thailand indicate that over 40.0% of the population experience insomnia or insufficient sleep, with approximately 10.0% affected by chronic insomnia [10].

In Thailand, the population aged ≥ 60 years is projected to rise to 20.2 million by 2025 [11]. In Phayao Province, northern Thailand, older adults accounted for approximately 26.1% of the population in 2023 [12]. This demographic transition has important implications for sleep health, as older adults are vulnerable to sleep disturbances due to age-related physiological changes, including neuronal degeneration and reduced function of sleep-regulating mechanisms [10]. Studying this highland, rural region of Northern Thailand is particularly crucial due to its unique socio-demographic and environmental challenges. Traditional agrarian lifestyles common in Phayao require demanding family and agricultural labor, leading to chronic physical fatigue, while the mountainous geography often limits timely access to specialized geriatric care and sleep medicine compared to urban centers [13]. In addition, older adults in rural communities often experience multiple contextual risk factors, such as chronic diseases, psychosocial stress, and environmental disturbances, which may contribute to poor sleep quality [12]. In Pha Chang Noi subdistrict, the expansion of tourism has introduced environmental changes, including localized noise emissions [14]. Although not a primary focus or measured variable in the present study, these environmental shifts highlight the changing landscape that may indirectly influence the living conditions of the older population [15].

Non-communicable diseases (NCDs) remain a major global health challenge and are the leading causes of disability and mortality, particularly among older adults [16]. A growing body of evidence indicates that sleep disturbances are closely associated with NCDs [17]. Conditions such as insomnia, sleep apnea, and short sleep duration have been identified as important correlates of cardiometabolic disorders, including diabetes mellitus (DM), hypertension (HT), and cardiovascular diseases, as well as mental health conditions [18]. Importantly, due to the cross-sectional nature of many studies, including the present study, these relationships should be interpreted as associations rather than causal effects. Older adults with pre-existing NCDs may experience poorer sleep due to disease-related symptoms, medication effects, or psychological burden, while poor sleep may also exacerbate disease severity, suggesting a bidirectional relationship [19]. This bidirectional relationship complicates the interpretation of findings from cross-sectional studies because sleep disturbances may function as both a potential contributor to and a consequence of chronic diseases. Therefore, the present study was designed to examine associations between sleep quality and health-related clinical outcomes and biomarkers rather than to establish causal relationships.

From an integrative health perspective, Traditional Chinese Medicine (TCM) associates insomnia with age-related physiological imbalances that conceptually overlap with chronic metabolic conditions [20,21]. Since this study was conducted in a region characterized by rich cultural diversity, incorporating TCM viewpoints, though not formally evaluated herein, provides a valuable complementary framework that reflects the diverse health beliefs and holistic care practices in the study area.

Rural Northern Thailand represents a distinctive setting for investigating sleep quality and health-related outcomes among older adults. Compared with urban areas, rural communities in northern Thailand have a higher proportion of older adults, many of whom rely on agriculture as their primary occupation and experience unique socioeconomic and environmental conditions. Access to specialized healthcare services is often limited because of mountainous geography and long travel distances to secondary or tertiary healthcare facilities. Consequently, the management of chronic diseases largely depends on primary healthcare services and community health volunteers. In addition, lifestyle characteristics, including early work schedules, physically demanding agricultural activities, and strong family-based caregiving, may influence both sleep quality and chronic disease management. These contextual factors may modify the relationship between sleep quality and health-related clinical outcomes and biomarkers, underscoring the importance of generating evidence from this specific population rather than extrapolating findings from urban or other regional settings. Therefore, this setting deserves separate investigation.

Despite the increasing burden of insomnia and NCDs, research integrating sleep quality, objective health-related biomarkers (e.g., blood pressure), and complementary medical perspectives remains limited in rural northern Thailand. Given these unique demographics, environmental, and healthcare characteristics, this study aimed to assess the association between sleep quality and health-related clinical outcomes and biomarkers among older adults in rural Northern Thailand. Although previous studies have demonstrated associations between sleep quality and individual cardiometabolic conditions, several important knowledge gaps remain. First, few studies have simultaneously examined multiple health-related clinical outcomes and biomarkers within the same population of community-dwelling older adults. Second, evidence from rural Northern Thailand is scarce despite the region’s distinctive demographic, environmental, and healthcare characteristics. Consequently, it remains unclear whether findings from urban populations or other regions can be generalized to this setting. Addressing these knowledge gaps is essential for developing context-specific interventions to promote healthy ageing and improve chronic disease prevention in rural communities. By integrating clinical and public health perspectives, this study seeks to provide context-specific evidence to support healthy aging strategies and inform public health policy.

## 2. Materials and Methods

### 2.1. Study Design and Study Period

A cross-sectional design was adopted. Data from a community-based research project were used, and a multi-stage cluster sampling strategy was utilized to select the participants. Phayao Province was purposively selected due to its rapidly growing older adult population and predominantly mountainous and remote geography, which restricts access to secondary health services, often requiring residents to travel 20–30 km for medical care. Of the nine districts in Phayao, Pong District was selected as the study site through the balloting method of selection. Subsequently, Phachangnoi Subdistrict was selected from the seven subdistricts of Pong District. Finally, three villages within Phachangnoi—Ban Pang Kha Tai, Ban Pang Prik, and Ban Pang Kha Nuea—were randomly selected for the study.

### 2.2. Study Setting, Population, and Sampling

The minimum sample size was determined using Daniel’s formula [22] for a single proportion at a 95% confidence level (CI) (Z = 1.96 Z = 1.96), a 5% margin of error, and an expected population proportion of 0.5, yielding a baseline requirement of *n* = 384. To account for potential data loss and to facilitate an equal 1:1 ratio for subgroup comparisons, the sample size was increased by approximately 10% and rounded up, resulting in a final sample of 424 participants (212 with NCDs and 212 without NCDs). A multistage sampling strategy was implemented. First, target villages within the Phachangnoi Subdistrict were selected using a simple random sampling technique (the lottery method). Subsequently, a quota sampling approach was applied at the individual level to recruit equal numbers of older adults with and without NCDs within the selected villages. This approach was chosen to ensure adequate representation of both subgroups and to provide sufficient statistical power for comparing sleep quality and health-related clinical outcomes between participants with and without NCDs. Because the primary objective of this study was to examine associations between sleep quality and health-related clinical outcomes rather than to estimate the population prevalence of NCDs, balanced recruitment of the two groups was considered appropriate. Accordingly, the reported prevalence of NCDs, hypertension, diabetes mellitus, and other health-related clinical outcomes in this study reflects the composition of the study sample and should not be interpreted as representing the epidemiological distribution of these conditions among older adults in rural Northern Thailand. However, because quota sampling is a non-probability sampling technique, the study sample may not fully represent the broader population of older adults in rural Northern Thailand. Consequently, the external validity and generalizability of the findings should be interpreted with appropriate caution. Eligible older adults were recruited through community announcements coordinated by village leaders, village health volunteers, and healthcare personnel at the Ban Pang Kha Subdistrict Health Promoting Hospital. Individuals who expressed interest were screened according to the predefined inclusion and exclusion criteria. Recruitment continued until the predetermined sample size, calculated using Daniel’s formula and implemented through quota sampling, was achieved. Eligible older adults were invited to participate in the study. Inclusion criteria were: (a) male or female individuals aged ≥60 years; (b) residency in Phachangnoi Subdistrict for at least one year; (c) ability to communicate in the local language; and (d) provision of written informed consent. Exclusion criteria included clinically diagnosed cognitive or psychiatric disorders, dementia, blindness, or other severe medical conditions.

### 2.3. Data Collection

Data were collected between April and October 2025. Before data collection, 10 local research assistants were recruited for each study area, comprising two public health professionals or nurses and eight village health volunteers. All assistants were long-term residents, fluent in the local language, and well-acquainted with the community, which facilitated efficient recruitment and participant engagement. Prior to data collection, research assistants participated in a two-hour training session covering the study objectives, data collection procedures, interview protocols, and standardized administration of the questionnaire to ensure accuracy and consistency. The training also addressed interview scheduling and emphasized ethical considerations, particularly the protection of participants’ rights. Data collection took place at the Ban Pang Kha Subdistrict Health Promoting Hospital between 09:00 and 16:00 h. Research assistants conducted face-to-face interviews using a structured questionnaire, with each session lasting approximately 10–15 min. Participants received a small token of appreciation upon completion. All questionnaires were reviewed daily by the research team, and incomplete responses were verified or rescheduled for follow-up the next day.

Blood pressure (BP) was measured using a calibrated digital sphygmomanometer under standardized conditions. Participants were seated and allowed to rest for at least 5 min prior to measurement. Blood pressure was recorded three times at 1–2 min intervals, and the average of the three readings was used for analysis to improve accuracy and reduce measurement error. For analytical purposes, elevated systolic blood pressure (SBP ≥ 130 mmHg) and elevated diastolic blood pressure (DBP ≥ 85 mmHg) were defined using the harmonized metabolic syndrome criteria proposed by the Joint Interim Statement, rather than hypertension diagnostic criteria [23]. These thresholds were used to represent cardiometabolic risk biomarkers rather than to establish a clinical diagnosis of hypertension. This approach was considered appropriate because the primary objective of the present study was to evaluate associations between sleep quality and cardiometabolic risk rather than to estimate the prevalence of hypertension. The harmonized metabolic syndrome criteria identify blood pressure levels associated with increased cardiometabolic risk and are widely used in epidemiological studies investigating metabolic abnormalities. Therefore, these thresholds provide a clinically meaningful framework for assessing cardiometabolic risk biomarkers among community-dwelling older adults. Fasting blood sugar (FBS) was measured following standard clinical procedures after an overnight fast of at least 8 h. Blood samples were collected by trained healthcare personnel and analyzed using standard laboratory methods.

### 2.4. Research Instruments

The contents of the interview instrument were validated by a panel of three experts in internal medicine, public health, and TCM to ensure relevance and appropriateness for use among older adults in the study setting. The questionnaire consisted of two sections. Section 1 collected demographic and health-related information, including age, gender, marital status, education, occupation, income, income sufficiency, sleep quality, alcohol consumption, smoking status, caffeine intake, underlying diseases, blood pressure (BP; measured at the time of the interview), and fasting blood sugar (FBS; assessed after at least eight hours of fasting, with water permitted). Section 2 assessed sleep quality using the Thai version of the PSQI, which was validated by Jirapramukpitak (1997) [24]. The PSQI is a self-reported instrument that evaluates sleep quality and disturbances over the past month. It comprises 18 items grouped into seven components: subjective sleep quality, sleep latency, sleep duration, habitual sleep efficiency, sleep disturbances, use of sleep medication, and daytime dysfunction. Each component is scored from 0 to 3, and the composite score from item summation ranges between 0 and 21. A score greater than 5 indicates poor sleep quality [24,25]. The Thai version of the PSQI was piloted among 30 individuals from the target population to evaluate its content validity and internal consistency. Content validity was assessed by three experts using the item–objective congruence (IOC) method, yielding an IOC value of 0.83, indicating acceptable content validity. The pilot study demonstrated good internal consistency, with a Cronbach’s alpha coefficient of 0.85. In the present study, the PSQI demonstrated acceptable internal consistency, with a Cronbach’s alpha coefficient of 0.71.

### 2.5. Statistical Analysis

The data were analyzed using IBM SPSS Statistics version 26.0 (IBM Corp., Armonk, NY, USA). Descriptive statistics were used to summarize participant characteristics and study variables. Categorical variables were presented as frequencies and percentages, whereas continuous variables were summarized as means ± standard deviations (SDs). Normality of continuous variables was assessed using the Kolmogorov–Smirnov test. Associations between sleep quality and categorical variables were initially examined using the Chi-square test or Fisher’s exact test, as appropriate. Multivariable binary logistic regression analysis was subsequently performed to estimate adjusted odds ratios (AORs) and 95% confidence intervals (CIs) for the associations between sleep quality and health-related clinical outcomes. Outcome variables were dichotomized using established clinical cut-off values, including those for fasting blood sugar and blood pressure, to facilitate clinically meaningful interpretation and ensure consistency with accepted clinical practice and previous epidemiological studies. For blood pressure, the cut-off values followed the Joint Interim Statement harmonized metabolic syndrome criteria because the analyses focused on cardiometabolic risk biomarkers rather than hypertension diagnosis. Categorical independent variables were entered into the models using dummy coding, with the lowest-risk or most favorable category serving as the reference group. All multivariable models were adjusted for sex, age, education, income sufficiency, alcohol consumption, smoking status, and caffeine intake.

Sparse data and separation issues were observed in the original analyses when the overall PSQI score was dichotomized into poor and good sleep quality groups, resulting in imprecise maximum-likelihood estimates and wide confidence intervals. Therefore, the overall PSQI score was modelled as a continuous variable to preserve the variability of the original score and reduce sparse-data problems associated with dichotomization. The PSQI component variables were analyzed according to their original categorical classifications. Although penalized likelihood methods, such as Firth logistic regression, may be considered in the presence of sparse data or separation, conventional logistic regression was retained because this approach provided more stable parameter estimates. Multicollinearity was assessed using variance inflation factors (VIFs), and no evidence of problematic multicollinearity was identified (all VIFs ≤ 1.5). Model fit was evaluated using the Hosmer–Lemeshow goodness-of-fit test, which indicated an adequate fit of the models. A two-sided *p*-value < 0.05 was considered statistically significant.

## 3. Results

Poor sleep quality (PSQI > 5) was observed in 29.7% of the participants. The mean PSQI score was 8.03 ± 4.86, with scores ranging from 1 to 17. Half of the participants (50.0%) reported at least one underlying condition, most commonly HT (35.4%), DM (17.5%), and other NCDs (14.6%), including chronic kidney disease, stroke, coronary artery disease, and hyperlipidemia. Because participants with and without non-communicable diseases were intentionally recruited in equal numbers using quota sampling, the reported proportions of HT, DM, and other NCDs reflect the composition of the study sample and should not be interpreted as prevalence estimates for older adults in rural Northern Thailand. About 21.0% of the participants had abnormal FBS (≥126 mg/dL), with a mean of 116.33 ± 30.84 mg/dL. Elevated SBP (≥130 mmHg) was observed in 34.0% of the participants, with a mean of 127.52 ± 12.01 mmHg. Elevated DBP (≥85 mmHg) was reported in 18.2% of participants, with a mean DBP of 75.85 ± 8.19 mmHg. These findings are presented in Table 1.

Chi-square analysis demonstrated significant associations between sleep quality and age, marital status, education, income sufficiency, caregiver status, having children, and caffeine consumption (tea/coffee). Similarly, analysis of health status indicated significant associations between the presence of underlying diseases and age, marital status, education, income sufficiency, and caffeine consumption. Marital status, education, and caffeine consumption (tea/coffee) were significantly associated with HT. Gender, age, occupation, family status, alcohol consumption, and smoking were significantly associated with DM (*p* < 0.05). Other NCDs (including chronic kidney disease, stroke, coronary artery disease, and hyperlipidemia) showed significant associations with income sufficiency, access to health information, and support from health personnel.

Analysis revealed significant associations between blood glucose levels and sex, age, education, occupation, income adequacy, alcohol consumption, smoking, and caffeine intake (tea/coffee). Education, and caffeine intake were associated with SBP. In addition, sex, education, and caffeine intake were significantly associated with DBP (*p* < 0.05), as presented in Table 2.

### Health-Related Clinical Variables Categorized by Sleep Quality Components 

The sample consisted of 424 participants, the majority of whom were female (58.0%), with a mean age of 67.5 years (SD = 5.4). More than half were married (66.5%), had completed some level of education (59.7%), were employed (65.1%), and reported a monthly income below 5000 THB (160 USD) (64.4%). A majority also reported insufficient income (59.9%), and more than half had received health-related information (51.2%), most commonly from their caregivers and children. Most participants did not consume alcohol (76.4%) or smoke (81.8%), while more than half consumed caffeine (56.8%). Chi-square analysis demonstrated significant associations between multiple components of sleep quality and health-related clinical variables. Overall sleep quality, subjective sleep quality, sleep duration, and habitual sleep efficiency were each significantly associated with disease status, HT, DM, NCDs, FBS, SBP, and DBP. (*p* < 0.05), as summarized in Table 3.

Multivariable logistic regression analyses demonstrated that each one-point increase in the overall PSQI score was significantly associated with higher odds of adverse health-related clinical outcomes and cardiometabolic biomarkers after adjustment for potential confounders. Higher PSQI scores were consistently associated with increased odds of underlying diseases, hypertension, diabetes mellitus, other non-communicable diseases, abnormal fasting blood sugar, elevated systolic blood pressure, and elevated diastolic blood pressure. Similar associations were observed for several individual sleep quality components, although some associations were not statistically significant after multivariable adjustment Table 4.

## 4. Discussion

This study aimed to assess the association between sleep quality and health-related clinical outcomes and biomarkers among older adults in rural Northern Thai communities and found a significant association between sleep quality and NCDs in this population. Approximately 29.7% of the participants reported poor sleep quality (PSQI > 5), with the highest prevalence observed among those with existing NCDs. These findings demonstrate significant associations between overall sleep quality and health-related clinical outcomes. Multivariable analyses further demonstrated that each one-point increase in the overall PSQI score was associated with increased odds of several adverse health-related clinical outcomes and cardiometabolic biomarkers after adjustment for potential confounders. However, because of the cross-sectional design, these associations should not be interpreted as causal relationships. Instead, the findings are consistent with a bidirectional relationship in which poor sleep and chronic diseases may mutually influence one another. Although sparse data and quasi-complete separation may have affected the precision of some regression estimates, the observed associations remained generally consistent and should therefore be interpreted with appropriate caution. Greater emphasis should be placed on the direction and consistency of the observed associations rather than on the magnitude of the estimated odds ratios. Furthermore, because participants with and without NCDs were intentionally recruited in equal numbers using quota sampling, the reported prevalence of NCDs, hypertension, diabetes mellitus, and other health-related clinical outcomes reflects the composition of the study sample rather than the epidemiological distribution of these conditions among older adults in rural Northern Thailand. Although this sampling strategy strengthened comparisons between groups, it may also have influenced the estimated odds ratios through selection bias. Accordingly, these findings should also be interpreted in light of the potential influence of selection bias introduced by quota sampling. Nevertheless, the observed associations are biologically plausible and may be explained by physiological mechanisms, including metabolic dysregulation, chronic inflammation, and autonomic imbalance. Conversely, older adults with established chronic diseases may also experience poorer sleep quality because of disease-related symptoms, including nocturia, chronic pain, medication adverse effects, and psychological distress. Therefore, the observed relationships are likely to be bidirectional, and the present cross-sectional design cannot determine the temporal sequence or causal direction of these associations. It is noteworthy that not all dimensions of sleep quality were significantly associated with every health-related clinical outcome. In particular, habitual sleep efficiency was not significantly associated with other non-communicable diseases after multivariable adjustment, and sleep duration showed no significant association with elevated diastolic blood pressure. These findings suggest that different components of sleep quality may influence specific clinical outcomes through distinct physiological mechanisms rather than exerting uniform effects across all cardiometabolic conditions. Overall and subjective sleep quality appeared to demonstrate more consistent associations across multiple outcomes, whereas habitual sleep efficiency and sleep duration may be more closely related to selected health indicators. Future longitudinal studies incorporating objective sleep assessments are warranted to further clarify these differential relationships. Consistent with previous reviews, insufficient or poor-quality sleep can activate the hypothalamic–pituitary–adrenal (HPA) axis, leading to elevated cortisol levels throughout the day. This dysregulation may induce low-grade chronic inflammation and impair metabolic control, including reduced insulin sensitivity and impaired glucose regulation, which is associated with increased odds of DM, HT, and cardiovascular disease [26,27]. Recent evidence further suggests that the relationship between sleep quality and cardiometabolic health is mediated through complex brain–body pathways that extend beyond activation of the hypothalamic–pituitary–adrenal (HPA) axis. Psychological distress, including chronic stress, anxiety, and depressive symptoms, may alter autonomic nervous system activity, neuroendocrine regulation, inflammatory responses, and metabolic homeostasis. These interconnected mechanisms contribute to glucose dysregulation, blood pressure elevation, and chronic low-grade inflammation, while metabolic abnormalities may in turn exacerbate sleep disturbances, supporting a bidirectional relationship between neurological health, sleep, and systemic metabolism. Although psychological distress was not directly assessed in the present study, these mechanisms provide a biologically plausible explanation for the observed associations between poor sleep quality and adverse clinical outcomes among older adults. This interpretation is consistent with recent evidence highlighting multimodal brain–body pathways linking psychological distress to metabolic dysfunction [28]. Recent neuroimaging evidence further suggests that internalizing psychopathology may also be associated with structural brain adaptations, including alterations in cortical surface area and cortical thickness, which may reflect underlying neurodevelopmental vulnerability and resilience. These structural changes may contribute to the complex brain–body mechanisms linking psychological distress with sleep disturbance and cardiometabolic dysfunction, although such mechanisms were not directly evaluated in the present study [29]. These findings should also be considered in light of potential residual confounding. Although the multivariable models were adjusted for several important sociodemographic and lifestyle characteristics, other clinically relevant factors, including body mass index, physical activity, obstructive sleep apnea, chronic pain, medication use, and subclinical psychological symptoms such as depression and anxiety, were not assessed or included in the analyses. Because these factors may influence both sleep quality and cardiometabolic health, they could have contributed to the observed associations. The present findings should also be interpreted within the context of the bidirectional relationship between sleep disturbances and chronic diseases. Although poor sleep may contribute to metabolic dysregulation, autonomic dysfunction, and systemic inflammation, chronic conditions such as hypertension and diabetes may likewise impair sleep quality through disease-related symptoms, treatment effects, psychological distress, and progressive physiological changes. Therefore, because of the cross-sectional design, the observed associations should not be interpreted as evidence of causality. Rather, the findings suggest a reciprocal relationship in which sleep disturbances and chronic diseases may influence one another over time.

Crucially, the duration of these NCDs likely plays a critical role; as this is a cross-sectional study, it is probable that a bidirectional relationship exists where long-term chronic illness further destabilizes sleep architecture over time. Although sparse data and quasi-complete separation may have reduced the precision of some regression estimates, the overall pattern of associations remained consistent. Therefore, greater emphasis should be placed on the direction and consistency of the observed associations rather than on the precise magnitude of individual effect estimates. 

An unexpected finding of this study was that a relatively higher proportion of participants with NCDs reported good sleep quality compared with those without NCDs. This observation should be interpreted with caution and may be explained by several factors. First, the study employed quota sampling to recruit equal numbers of participants with and without NCDs rather than a population-based sampling strategy. Consequently, the distribution of sleep quality may not reflect that of the general older adult population. Second, many participants with chronic diseases were receiving regular follow-up care at primary healthcare facilities, where they may have benefited from continuous disease management, health education, medication adherence, and lifestyle counseling, all of which could have contributed to improved sleep quality. Finally, survival bias, selection bias, and residual confounding cannot be excluded. Older adults with chronic diseases who were sufficiently healthy to participate in this community-based study may represent a relatively healthier subgroup of individuals living with chronic conditions. In addition, unmeasured lifestyle adaptations, such as improved dietary habits, sleep hygiene, or other health-promoting behaviors, may also have contributed to better sleep quality but were not assessed in the present study. Therefore, these findings should not be interpreted as indicating that older adults with NCDs generally experience better sleep quality than those without NCDs. 

From a theoretical perspective within Traditional Chinese Medicine (TCM), sleep quality is regarded as a manifestation of Yin–Yang balance and the coordinated function of the Heart, Liver, Kidney, and Spleen. Chronic insufficient sleep is thought to induce Kidney Yin Deficiency, which fails to regulate Heart Fire, leading to restlessness of the Shen (mind/spirit) and symptoms such as insomnia or frequent awakenings. Furthermore, inadequate control of Liver Fire may contribute to HT [30], while impaired liver function can disrupt blood storage and circulation, resulting in Qi and Blood stagnation. This theoretical interpretation parallels contemporary biomedical perspectives on organ function and metabolic regulation. From the perspective of TCM clinical theory, stagnation of Liver Qi disrupts the circulation of Qi and Blood, leading to excess Liver Fire that manifests as insomnia and HT. Additionally, Spleen Deficiency, which impairs Blood generation and regulation, can promote dampness and phlegm accumulation, obstructing the Heart Meridian and further disturbing sleep. These mechanisms are consistent with modern evidence showing that effective management of insomnia may improve metabolic profiles, including elevation of HDL-c, a recognized protective factor against cardiovascular disease [30,31]. These findings suggest that maintaining Yin–Yang balance and supporting the coordinated function of key organs may play a critical role in supporting efforts to prevent and manage NCDs among older adults. This interpretation is consistent with global evidence. A study of patients with chronic illness in Ethiopia reported that 52.0% experienced poor sleep quality, with prevalence varying by disease type—for example, 36.0% among individuals with diabetes [32]. Similarly, research in Taiwan found that 53.1% of participants with PSQI >5 had poor sleep quality [33]. In China, older adults with multimorbidity showed significantly higher PSQI scores and more frequent poor sleep quality compared to those without chronic conditions, with risk increasing proportionally with the number of comorbidities [34].

Our analysis of sociodemographic variables demonstrated that age, marital status, education, income adequacy, caregiver status, and caffeine consumption (tea/coffee) were significantly associated with sleep quality. These factors were also significantly related to the presence of underlying NCDs. The study demonstrated that older adults with HT, DM, chronic kidney disease, stroke, cardiovascular disease, and hyperlipidemia experienced significantly poorer sleep quality than those without chronic conditions. These findings are consistent with prior reviews suggesting that NCDs can disrupt sleep through multiple pathways, including chronic pain, autonomic dysfunction, sleep apnea, and low-grade systemic inflammation [35]. They also align with evidence that aging is associated with physiological changes in sleep architecture, such as reduced slow-wave sleep, increased light sleep, and more frequent nighttime awakenings that collectively contribute to diminished sleep quality [36]. Advancing age is strongly associated with higher risks of HT and DM, largely attributable to autonomic nervous system decline and reduced vascular elasticity [37].

In our study population, nearly half of the participants (40.3%) had no formal education or only primary schooling; the majority were farmers, more than half reported inadequate income, and approximately one-quarter smoked or consumed alcohol. These sociodemographic and lifestyle factors likely contribute to the elevated burden of chronic disease observed among older adults in rural areas. This is consistent with prior research suggesting that rural populations often undervalue healthy lifestyle practices and the importance of adequate sleep quality [38]. Socioeconomic factors, such as marital status and income adequacy, may contribute to chronic stress, thereby increasing vulnerability to sleep disturbances [39]. Chronic stress is known to activate the HPA axis, elevating cortisol levels and disrupting normal sleep regulation [40]. In addition, the absence of a caregiver may heighten psychological burden, further impairing sleep quality [41]. These findings are consistent with systematic evidence showing that caffeine consumption in the form of tea or coffee, particularly in the afternoon or evening, can inhibit adenosine signaling—a key neurotransmitter that promotes sleep—thereby reducing both sleep duration and quality [42]. Furthermore, recent studies have demonstrated significant associations between caffeine intake and adverse sleep-related health outcomes [43].

This study identified sex, age, education, occupation, sufficient income, alcohol consumption, smoking, and caffeine intake (tea/coffee) as significant predictors of FBS and SBP. These associations likely reflect the combined influence of biological and socioeconomic determinants on cardiometabolic health. Prior evidence indicates that women tend to have higher BP and blood glucose levels than men—a disparity that may be explained by the decline in estrogen following menopause. Reduced estrogen contributes to decreased vascular elasticity, diminished insulin sensitivity, and consequently a higher risk of HT and DM [44,45]. Previous reviews highlight that behavioral risk factors such as alcohol consumption, smoking, and caffeine intake activate the sympathetic nervous system and promote inflammation, thereby increasing SBP and blood glucose levels [39,43,46]. From a TCM perspective, these pathophysiological changes correspond to Kidney Yin Deficiency, commonly observed after middle age. In TCM theory, the Kidney stores essential essence and regulates the Yin–Yang balance of the body [43,46]. Deficiency of Kidney Yin leads to excessive internal heat, resulting in hyperactive Kidney Yang that disrupts the Heart and Liver. The resulting excess Heart and Liver Fire impairs the circulation of Qi and Blood, manifesting as palpitations, tachycardia, insomnia, and dizziness, which may further contribute to elevated BP and hyperglycemia [30,31].

This study found that sleep quality was significantly associated with underlying conditions, including HT, DM, other NCDs, FBS, SBP, and DBP. These associations may be attributed to the physiological stress and chronic low-grade inflammation commonly observed in individuals with such conditions. These factors can disrupt the secretion of key sleep-regulating hormones, particularly melatonin and cortisol, thereby disturbing circadian rhythms and reducing overall sleep quality [47,48]. Our findings align with a systematic review from China showing that 52.5% of patients with HT reported poor sleep quality—a prevalence 2.66 times higher than in non-hypertensive controls [49]. Consistent evidence from multiple studies further indicates that both HT and DM are closely associated with insufficient or poor-quality sleep, which may exacerbate metabolic dysfunction and increase the risk of complications such as elevated blood glucose [32,35,50,51]. In parallel with these clinical perspectives, TCM literature proposes that sleep disturbances may be conceptually associated with deficiency syndromes, particularly Yin Deficiency and Yang Deficiency, which impair the circulation of Qi and Blood. Empirical studies further indicate that sleep disturbances are associated with Qi Deficiency, Yin Deficiency, Blood Stasis, and Qi Stagnation constitutions [31]—all of which have been linked to an elevated risk of sleep disorders [52]. Collectively, these findings highlight the importance of evaluating sleep quality in patients with chronic diseases as part of a comprehensive and integrated approach to management.

Analysis of PSQI components indicated that subjective sleep quality, sleep duration, and habitual sleep efficiency were significantly associated with HT, DM, other NCDs, and clinical measures including FBS, SBP, and DBP. These associations were most pronounced among older adults aged 65–69 years—an age group characterized by physical and psychological transitions that can negatively affect sleep quality and duration [53]. Poor sleep in this age group has been linked to fatigue, cognitive impairment, physical and psychological decline, and diminished quality of life [39,43,54]. Sleep disturbances not only complicate the management of chronic diseases but also contribute to increased social disability [39,54]. Systematic reviews have shown that both short and long sleep durations are associated with a greater risk of HT [55], impaired glycemic control, and type 2 diabetes [56]. Epidemiological evidence further indicates that nearly half of the population reports sleep problems, with prevalence particularly high among individuals with DM, HT, and cardiovascular disease [55]. From a TCM perspective, disordered sleep reflects imbalances in Qi, Jing, and Shen—most notably disharmony between the Heart and Kidney and stagnation of Liver Qi—which may help explain the observed associations with HT and abnormal glucose metabolism [49].

Multivariable logistic regression analyses demonstrated that higher overall PSQI scores were significantly associated with underlying diseases, hypertension, non-communicable diseases, abnormal fasting blood sugar, elevated systolic blood pressure, and elevated diastolic blood pressure among older adults. Specifically, each one-point increase in the overall PSQI score was associated with increased odds of adverse health-related clinical outcomes after adjustment for potential confounding factors. These findings suggest that poorer sleep quality may be associated with a greater burden of chronic diseases and unfavorable cardiometabolic health among older adults. Conversely, better sleep quality may contribute to more favorable health outcomes and a lower burden of non-communicable diseases in ageing populations [55]. Evidence from previous reviews highlights that high-quality sleep is essential for daily physiological restoration and is associated with immune function, metabolic regulation, endocrine activity, and anti-inflammatory responses [55,57,58]. These findings are also broadly consistent with Traditional Chinese Medicine (TCM), which considers good-quality sleep an indicator of physiological balance involving the harmonious interaction of Qi, Jing, and Shen [43,46]. Age-related decline in Kidney Qi and Kidney essence has been proposed to contribute to sleep disturbances and reduced physiological resilience in older adults [20,46,49]. Although TCM-related variables were not assessed in the present study, this perspective provides complementary background for understanding potential mechanisms linking sleep quality and healthy ageing.

Analysis of PSQI components showed that older adults with poorer subjective sleep quality had significantly higher risks of underlying diseases, HT, NCDs, FBS, SBP, and DBP compared with those reporting very good sleep quality. These results indicate that perceived sleep quality is closely associated with multiple dimensions of health. Contributing factors may include difficulties in maintaining healthy lifestyles, limited awareness about the importance of sleep and the consequences of its lack thereof, and risk behaviors such as alcohol consumption, smoking, and caffeine intake, all of which negatively affect sleep quality and overall health [39,42]. Consistent with previous studies, poor subjective sleep quality has been associated with adverse health outcomes [59] and demonstrates a significant relationship with NCDs [58].

Analysis of sleep duration showed that shorter sleep duration was associated with higher odds of underlying diseases, HT, NCDs, elevated FBS, SBP, and DBP compared with sleep duration of more than 7 h. These findings align with evidence that insufficient sleep impairs metabolic regulation, promotes inflammation, and disrupts glycemic control—key mechanisms underlying the development of multiple chronic diseases [53]. Consistent with previous research, sleeping fewer than 5 h has also been shown to exert particularly detrimental effects on health [59]. These findings are consistent with evidence that inadequate sleep duration is associated with increased mortality risk among patients with NCDs [60]. A study from Korea similarly demonstrated that, compared with individuals sleeping 6–8 h per day, those with shorter sleep duration had a significantly higher risk of mortality and poorer health-related quality of life [61].

Our analysis revealed that older adults with habitual sleep efficiency of 65.0–74.0% had significantly higher risks of underlying diseases, HT, NCDs, FBS, abnormal SBP, and DBP. These findings are consistent with prior reviews showing that reduced sleep efficiency is a common feature among individuals with chronic pain, illness-related anxiety, and fragmented sleep, underscoring its role as a key marker of impaired sleep quality [53,59,62]. Chronic conditions and NCDs have been identified as reliable predictors of reduced sleep efficiency [59,63]. Consistent with our findings, prior research has shown that older adults with sleep efficiency below 80.0% face a significantly higher risk of mortality [64]. Moreover, evidence indicates that individuals with sleep efficiency of around 65% experience significant adverse effects on health, with poor efficiency serving as a strong predictor of physical health, mental well-being, and cognitive function [58,59].

Taken together, these findings suggest that subjective sleep quality, sleep duration, and habitual sleep efficiency are the PSQI components with the greatest clinical relevance in this study. Among these, subjective sleep quality demonstrated the most consistent associations across multiple health-related clinical outcomes and biomarkers, indicating that an individual’s overall perception of sleep may serve as an important indicator of underlying health status. Sleep duration and habitual sleep efficiency also showed consistent associations with cardiometabolic outcomes, supporting previous evidence that insufficient sleep and fragmented sleep contribute to impaired glucose regulation, blood pressure dysregulation, and chronic disease burden. These findings suggest that these specific dimensions of sleep quality may represent practical targets for routine screening and early intervention among older adults in primary healthcare settings.

However, several methodological considerations should be noted when interpreting these findings. The very large odds ratios observed in this study should be interpreted with caution, as these estimates may be influenced by sparse data and potential complete or quasi-complete separation in some variables, particularly where certain conditions were observed exclusively in one outcome category. Notably, diabetes mellitus could not be reliably estimated in the multivariable logistic regression analysis due to complete separation, as all participants with diabetes were classified as having poor sleep quality. This issue may lead to unstable or inflated parameter estimates and limits the interpretability of the corresponding odds ratios. Despite these limitations, the observed patterns suggest a strong association between sleep quality and adverse health outcomes, including diabetes mellitus. While the direction of the associations remains consistent with prior evidence, the magnitude of the odds ratios may be overestimated. Furthermore, the absence of longitudinal data regarding the precise duration of NCDs and the onset of sleep impairments remains a notable limitation, as the chronicity of illness may significantly modulate these clinical outcomes. Future studies using larger and more balanced samples, or alternative analytical approaches such as penalized logistic regression, are recommended to obtain more stable and reliable estimates.

## 5. Conclusions

This study found significant associations between higher overall PSQI scores and NCDs, as well as several health-related clinical outcomes and biomarkers, among older adults in rural Northern Thailand. Nearly one-third of participants reported poor sleep quality, with a higher prevalence observed among those with chronic conditions. Higher overall PSQI scores, together with shorter sleep duration and lower habitual sleep efficiency, were associated with adverse health indicators, including elevated blood glucose and blood pressure levels. These findings highlight the importance of incorporating sleep quality assessment into routine health screening and chronic disease management programs for older adults. Community-based health promotion strategies that support healthy sleep behaviors and address modifiable lifestyle factors, such as alcohol consumption, smoking, and caffeine intake, may contribute to improved health outcomes. In addition, strengthening support from caregivers, community health workers, and local health services may help promote sustainable sleep health among older adults in rural communities. Given the cross-sectional design of this study, causal relationships cannot be established. Future longitudinal studies using objective sleep measurements and intervention studies are warranted to further clarify the temporal and potentially bidirectional relationships between sleep quality and chronic health conditions among older adults.

## 6. Limitations and Recommendations for Future Research

This study has several limitations that should be considered when interpreting the findings. First, the cross-sectional design precludes any inference of causal relationships between sleep quality and health-related clinical outcomes and biomarkers. Although significant associations were observed, the temporal direction of these relationships cannot be established. Therefore, the findings should be interpreted as evidence of associations rather than causation, particularly given the likely bidirectional relationship between sleep disturbances and chronic diseases. Second, sleep quality was assessed using the self-reported Pittsburgh Sleep Quality Index (PSQI), which, although widely validated, relies on participants’ recall of sleep experiences during the preceding month. Among older adults, age-related changes in memory and cognitive function may reduce the accuracy of self-reported sleep characteristics, thereby introducing recall and reporting bias. Furthermore, subjective assessments of sleep quality do not always correspond with objective physiological measures obtained from polysomnography or actigraphy. Consequently, the prevalence of poor sleep quality reported in this study may differ from objectively measured sleep disturbances. Future studies should incorporate objective sleep assessments together with longitudinal study designs to improve the accuracy of sleep measurement and clarify the temporal relationships between sleep quality and health-related clinical outcomes. Third, fasting blood sugar, systolic blood pressure, and diastolic blood pressure were categorized using established clinical cut-off values to facilitate clinically meaningful interpretation and logistic regression analyses. In contrast, the overall PSQI score was analyzed as a continuous variable to preserve the full range of information and minimize the loss of statistical information associated with dichotomization. Nevertheless, categorizing clinical outcome variables may have reduced statistical power and obscured potentially important variability within the data. Future studies should further evaluate these associations using alternative statistical approaches where appropriate and examine both continuous and categorical outcome measures. Fourth, although participants with clinically diagnosed cognitive or psychiatric disorders were excluded, several potentially important confounding factors were not assessed, including body mass index (BMI), obstructive sleep apnea or habitual snoring, chronic pain, medication use and polypharmacy (particularly hypnotic, antihypertensive, and antidiabetic medications), physical activity, subclinical psychological symptoms (e.g., mild depressive symptoms and chronic anxiety), and the duration of underlying non-communicable diseases. The chronicity of conditions such as hypertension and diabetes mellitus may influence both sleep quality and cardiometabolic health through cumulative physiological changes over time. Because these variables were unavailable, residual confounding cannot be excluded, and the observed associations should therefore be interpreted with caution. Future studies should incorporate these clinically relevant variables into multivariable models to obtain more precise estimates of the relationships between sleep quality and health-related clinical outcomes. Fifth, although the overall regression estimates were generally consistent, sparse data and quasi-complete separation in some outcome categories may have affected the precision of several regression estimates, resulting in wider confidence intervals and greater statistical uncertainty. Accordingly, greater emphasis should be placed on the direction and consistency of the observed associations rather than on the precise magnitude of the estimated odds ratios. Finally, quota sampling was employed to ensure balanced representation of participants with and without non-communicable diseases for comparative analyses. Although this approach strengthened comparisons between the study groups, it is a non-probability sampling method and may therefore have reduced the representativeness of the study sample. Consequently, the external validity and generalizability of the findings may be limited, particularly when extrapolating the results to older adults living in other rural or urban settings in Thailand. Furthermore, because participants with and without non-communicable diseases were intentionally recruited in equal numbers, the reported prevalence of chronic diseases should not be interpreted as representative of the broader population of older adults in rural Northern Thailand. This sampling strategy may also have influenced the estimated odds ratios through selection bias, and therefore the magnitude of the observed associations should be interpreted with appropriate caution. In addition, because quota sampling intentionally recruited equal numbers of participants with and without non-communicable diseases, the reported frequencies of NCDs, hypertension, diabetes mellitus, and other clinical outcomes reflect the study design rather than the underlying epidemiological distribution in the target population. Accordingly, these descriptive findings should not be interpreted as prevalence estimates for older adults in rural Northern Thailand. Future studies using probability-based sampling methods across multiple geographic regions are warranted to improve the representativeness and generalizability of the findings.

In addition, psychosocial, non-pharmacological interventions should be recommended, such as TCM practices and local herbal remedies, integrated with sleep health promotion to improve sleep quality in communities. Future research should expand to other regions and include a wider range of age groups to improve the generalizability of the findings. Third, biomarkers in this study, including blood glucose and blood pressure, were measured only once, which may not adequately capture long-term health status. Potential confounders that may have influenced the results as covariates—such as depression, anxiety, medication history, and other comorbidities—were not assessed. Information on the duration of diabetes was not collected in this study and should be considered in future research. Nevertheless, the study adjusted for key variables identified in the previous literature, which helped minimize bias and enhance the validity of the findings. 

## Figures and Tables

**Table 1:** Descriptive results of sleep quality (PSQI) and health-related clinical variables among the participants (*n* = 424).

Outcome	*n* (%)	Mean ± SD	Min–Max
Overall Pittsburgh Sleep Quality Index (PSQI)			
Poor sleep (>5 score)	126 (29.7%)	8.03 ± 4.86	1–17
Good sleep (≤5 score)	298 (70.3%)		
All NCDs (HT, DM, and other NCDs)			
No	212 (50.0%)		
Yes	212 (50.0%)		
Hypertension (HT)			
No	274 (64.6%)		
Yes	150 (35.4%)		
Diabetes Mellitus (DM)			
No	350 (82.5%)		
Yes	74 (17.5%)		
Other NCDs (CKD, Stroke, CAD, Hyperlipidemia)			
No	362 (85.4%)		
Yes	62 (14.6%)		
Fasting Blood Sugar (FBS)			
Normal (<126 mg/dL)	335 (79.0%)	116.33 ± 30.84	67–240
Abnormal (≥126 mg/dL)	89 (21.0%)		
Systolic Blood Pressure (SBP)			
Normal (<130 mmHg)	280 (66.0%)	127.52 ± 12.01	105–180
Abnormal (≥130 mmHg)	144 (34.0%)		
Diastolic Blood Pressure (DBP)			
Normal (<85 mmHg)	347 (81.8%)	75.85 ± 8.19	60–100
Abnormal (≥85 mmHg)	77 (18.2%)		

Abbreviations: CKD: Chronic kidney disease; CAD: Coronary artery disease.

**Table 2:** Sleep quality (PSQI) and health-related clinical variables of the participant categories and general characteristics (*n* = 424).

Variable	*n* (%)	Poor Sleep (*n* = 126)	Having NCDs (*n* = 212)	HT (*n* = 150)	DM (*n* = 74)	Other NCDs (*n* = 62)	Abnormal FBS (*n* = 89)	Abnormal SBP (*n* = 144)	Abnormal DBP (*n* = 77)
Gender		*p* = 0.189	*p* = 0.140	*p* = 0.841	*p* = 0.009 *	*p* = 0.161	*p* = 0.006 *	*p* = 0.461	*p* = 0.034 *
Male	178 (42.0%)	59 (33.1%)	81 (45.5%)	62 (34.8%)	21 (11.8%)	21 (11.8%)	26 (14.6%)	64 (36.0%)	24 (13.5%)
Female	246 (58.0%)	67 (27.2%)	131 (53.3%)	88 (35.8%)	53 (21.5%)	41 (16.7%)	63 (25.6%)	80 (32.5%)	53 (21.5%)
Age		*p* = 0.042 *	*p* = 0.009 *	*p* = 0.166	*p* = 0.032 *	*p* = 0.055	*p* = 0.002 *	*p* = 0.050	*p* = 0.222
60–64 years	129 (30.4%)	48 (37.2%)	52 (40.3%)	37 (28.7%)	17 (13.2%)	13 (10.1%)	18 (14.0%)	33 (25.6%)	18 (14.0%)
65–69 years	180 (42.5%)	52 (28.9%)	91 (50.6%)	69 (38.3%)	28 (15.6%)	25 (13.9%)	34 (18.9%)	66 (36.7%)	33 (18.3%)
≥70 years	115 (27.1%)	26 (22.6%)	69 (60.0%)	44 (38.3%)	29 (25.2%)	24 (20.9%)	37 (32.2%)	45 (39.1%)	26 (22.6%)
Marital status		*p* = 0.013 *	*p* = 0.050 *	*p* = 0.041 *	*p* = 0.176	*p* = 0.069	*p* = 0.118	*p* = 0.057	*p* = 0.183
Single/Widowed/ Divorced/Separated	142 (33.5%)	31 (21.8%)	81 (57.0%)	60 (42.3%)	30 (21.1%)	27 (19.0%)	36 (25.4%)	57 (40.1%)	31 (21.8%)
Married	282 (66.5%)	95 (33.7%)	131 (46.5%)	90 (31.9%)	44 (15.6%)	35 (12.4%)	53 (18.8%)	87 (30.9%)	46 (16.3%)
Education		*p* = 0.007 *	*p* < 0.001 *	*p* = 0.005 *	*p* = 0.119	*p* = 0.162	*p* = 0.027 *	*p* = 0.001 *	*p* < 0.001 *
No	171 (40.3%)	38 (22.2%)	106 (62.0%)	74 (43.3%)	36 (21.1%)	30 (17.5%)	45 (26.3%)	74 (43.3%)	47 (27.5%)
Yes	253 (59.7%)	88 (34.8%)	106 (41.9%)	76 (30.0%)	38 (15.0%)	32 (12.6%)	44 (17.4%)	70 (27.7%)	30 (11.9%)
Occupation		*p* = 0.435	*p* = 0.262	*p* = 0.457	*p* = 0.032 *	*p* = 0.918	*p* = 0.006 *	*p* = 0.709	*p* = 0.188
No	148 (34.9%)	40 (27.0%)	80 (54.1%)	56 (37.8%)	34 (23.0%)	22 (14.9%)	42 (28.4%)	52 (35.1%)	32 (21.6%)
Yes	276 (65.1%)	86 (31.2%)	132 (47.8%)	94 (34.1%)	40 (14.5%)	40 (14.5%)	47 (17.0%)	92 (33.3%)	45 (16.3%)
Income		*p* = 0.222	*p* = 0.310	*p* = 0.672	*p* = 0.790	*p* = 0.792	*p* = 0.502	*p* = 0.625	*p* = 0.513
<5000 Baht	273 (64.4%)	87 (31.9%)	142 (52.0%)	99 (36.3%)	49 (17.9%)	39 (14.3%)	60 (22.0%)	95 (34.8%)	47 (17.2%)
≥5000 Baht	151 (35.6%)	39 (25.8%)	70 (46.4%)	51 (33.8%)	25 (16.6%)	23 (15.2%)	29 (19.2%)	49 (32.5%)	30 (19.9%)
Sufficient income		*p* < 0.001 *	*p* = 0.004 *	*p* = 0.409	*p* = 0.090	*p* < 0.001 *	*p* = 0.035 *	*p* = 0.159	*p* = 0.898
Insufficient	254 (59.9%)	53 (20.9%)	142 (55.9%)	94 (37.0%)	51 (20.1%)	51 (20.1%)	62 (24.4%)	93 (36.6%)	47 (18.5%)
Sufficient	170 (40.1%)	73 (42.9%)	70 (41.2%)	56 (32.9%)	23 (13.5%)	11 (6.5%)	27 (15.9%)	51 (30.0%)	30 (17.6%)
Health information		*p* = 0.288	*p* = 0.560	*p* = 0.417	*p* = 1.000	*p* = 0.005 *	*p* = 0.729	*p* = 0.637	*p* = 0.707
No	207 (48.8%)	67 (32.4%)	100 (48.3%)	69 (33.3%)	36 (17.4%)	20 (9.7%)	42 (20.3%)	68 (32.9%)	36 (17.4%)
Yes	217 (51.2%)	59 (27.2%)	112 (51.6%)	81 (37.3%)	38 (17.5%)	42 (19.4%)	47 (21.7%)	76 (35.0%)	41 (18.9%)
-Staff		*p* = 0.067	*p* = 0.491	*p* = 0.306	*p* = 1.000	*p* = 0.005 *	*p* = 0.692	*p* = 0.909	*p* = 0.899
No	246 (58.0%)	82 (33.3%)	119 (48.4%)	82 (33.3%)	43 (17.5%)	26 (10.6%)	50 (20.3%)	83 (33.7%)	44 (17.9%)
Yes	178 (42.0%)	44 (24.7%)	93 (52.2%)	68 (38.2%)	31 (17.4%)	36 (20.2%)	39 (21.9%)	61 (34.3%)	33 (18.5%)
-Family		*p* = 1.000	*p* = 1.000	*p* = 0.904	*p* = 0.032 *	*p* = 0.050	*p* = 0.096	*p* = 0.694	*p* = 0.177
No	328 (77.4%)	97 (29.6%)	164 (50.0%)	117 (35.7%)	50 (15.2%)	42 (12.8%)	63 (19.2%)	113 (34.5%)	55(16.8%)
Yes	96 (22.6%)	29 (30.2%)	48 (50.0%)	33 (34.4%)	24 (25.0%)	20 (20.8%)	26 (27.1%)	31 (32.3%)	22 (22.9%)
Caregiver status		*p* = 0.007 *	*p* = 1.000	*p* = 1.000	*p* = 0.276	*p* = 0.063	*p* = 0.334	*p* = 0.979	*p* = 0.831
No	41 (9.7%)	20 (48.8%)	20 (48.8%)	15 (36.6%)	10 (24.4%)	2 (4.9%)	11 (26.8%)	14 (34.1%)	8 (19.5%)
Yes	383 (90.3%)	106 (27.7%)	192 (50.1%)	135 (35.2%)	64 (16.7%)	60 (15.7%)	78 (20.4%)	130 (33.9%)	69 (18.0%)
-Child		*p* = 0.029 *	*p* = 0.195	*p* = 0.836	*p* = 0.240	*p* = 0.160	*p* = 0.279	*p* = 0.721	*p* = 0.897
No	164 (67.0%)	59 (36.0%)	75 (45.7%)	57 (34.8%)	24 (14.6%)	19 (11.6%)	30 (18.3%)	54 (32.9%)	29 (17.7%)
Yes	260 (67.0%)	67 (25.8%)	137 (52.7%)	93 (35.8%)	50 (19.2%)	43 (16.5%)	59 (22.7%)	90 (34.6%)	48 (18.5%)
Alcohol consumption		*p* = 1.000	*p* = 0.567	*p* = 0.283	*p* = 0.002 *	*p* = 0.903	*p* = 0.002 *	*p* = 0.224	*p* = 0.557
No	324 (76.4%)	96 (29.6%)	165 (50.9%)	110 (34.0%)	67 (20.7%)	47 (14.5%)	79 (24.4%)	105 (32.4%)	61 (18.8%)
Yes	100 (23.6%)	30 (30.0%)	47 (47.0%)	40 (40.0%)	7 (7.0%)	15 (15.0%)	10 (10.0%)	39 (39.0%)	16 (16.0%)
Smoking		*p* = 0.169	*p* = 0.077	*p* = 0.600	*p* = 0.045 *	*p* = 0.245	*p* = 0.027 *	*p* = 0.968	*p* = 0.625
No	347 (81.8%)	98 (28.2%)	181 (52.2%)	125 (36.0%)	67 (19.3%)	54 (15.6%)	80 (23.1%)	118 (34.0%)	65 (18.7%)
Yes	77 (18.2%)	28 (36.4%)	31 (40.3%)	25 (32.5%)	7 (9.1%)	8 (10.4%)	9 (11.7%)	26 (33.8%)	12 (15.6%)
Caffeine (Tea/Coffee)		*p* < 0.001 *	*p* < 0.001 *	*p* < 0.001 *	*p* = 0.093	*p* = 0.061	*p* = 0.006 *	*p* < 0.001 *	*p* < 0.001 *
No	183 (43.2%)	76 (41.5%)	53 (29.0%)	31 (16.9%)	25 (13.7%)	20 (10.9%)	27 (14.8%)	31 (16.9%)	15 (8.2%)
Yes	241 (56.8%)	50 (20.7%)	159 (66.0%)	119 (49.4%)	49 (20.3%)	42 (17.4%)	62 (25.7%)	113 (46.9%)	62 (25.7%)

Notes: * *p*-value < 0.05; PSQI: Pittsburgh sleep quality index; HT: Hypertension; DM: Diabetes mellitus; NCDs: Non-communicable diseases; FBS: Fasting blood sugar; SBP: Systolic blood pressure; DBP: Diastolic blood pressure. Bold values indicate statistical significance (*p* < 0.05).

**Table 3:** Health-related clinical variables of the participant categories by sleep quality components (*n* = 424).

Variable	*n* (%)	All NCDs		HT		DM		Other NCDs		FBS		SBP		DBP	
		No (*n* = 212)	Yes (*n* = 212)	No (*n* = 274)	Yes (*n* = 150)	No (*n* = 350)	Yes (*n* = 74)	No (*n* = 362)	Yes (*n* = 62)	<126 mg/dL (*n* = 335)	≥126 mg/dL (*n* = 89)	<130 mmHg (*n* = 280)	≥130 mmHg (*n* = 144)	<85 mmHg (*n* = 347)	≥85 mmHg (*n* = 77)
Overall PSQI			*p* < 0.001		*p* < 0.001		*p* < 0.001		*p* < 0.001		*p* < 0.001		*p* < 0.001		*p* < 0.001
Poor sleep (>5 score)	126 (29.7%)	123 (97.6%)	3 (2.4%)	123 (97.6%)	3 (2.4%)	126 (100.0%)	0(0.0%)	126 (100.0%)	0(0.0%)	126 (100.0%)	0(0.0%)	123 (97.6%)	3 (2.4%)	124 (98.4%)	2 (1.6%)
Good sleep (≤5 score)	298 (70.3%)	89 (29.9%)	209 (70.1%)	151 (50.7%)	147 (49.3%)	224 (75.2%)	74 (24.8%)	236 (79.2%)	62 (20.8%)	209 (70.1%)	89 (29.9%)	157 (52.7%)	141 (47.3%)	223 (74.8%)	75 (25.2%)
Subjective sleep quality			*p* < 0.001 *^a^*		*p* < 0.001 *^a^*		*p* < 0.001 *^a^*		*p* < 0.001 *^a^*		*p* < 0.001 *^a^*		*p* < 0.001 *^a^*		*p* < 0.001 *^a^*
Very good	186 (43.9%)	158 (84.9%)	28 (15.1%)	165 (88.7%)	21 (11.3%)	178 (95.7%)	8 (4.3%)	178 (95.7%)	8 (4.3%)	174 (93.5%)	12 (6.5%)	166 (89.2%)	20 (10.8%)	177 (95.2%)	9 (4.8%)
Fairly bad	141 (33.3%)	39 (27.7%)	102 (72.3%)	72 (51.1%)	69 (48.9%)	106 (75.2%)	35 (24.8%)	107 (75.9%)	34 (24.1%)	99 (70.2%)	42 (29.8%)	74 (52.5%)	67 (47.5%)	106 (75.2%)	35 (24.8%)
Very bad	97 (22.9%)	15 (15.5%)	82 (84.5%)	37 (38.1%)	60 (61.9%)	66 (68.0%)	31 (32.0%)	77 (79.4%)	20 (20.6%)	62 (63.9%)	35 (36.1%)	40 (41.2%)	57 (58.8%)	64 (66.0%)	33 (34.0%)
Sleep duration			*p* < 0.001 *^a^*		*p* < 0.001 *^a^*		*p* = 0.001 *^a^*		*p* < 0.001 *^a^*		*p* < 0.001 *^a^*		*p* < 0.001 *^a^*		*p* = 0.055 *^a^*
>7 h	269 (63.4%)	171 (63.6%)	98 (36.4%)	199 (74.0%)	70 (26.0%)	236 (87.7%)	33 (12.3%)	254 (94.4%)	15 (5.6%)	231 (85.9%)	38 (14.1%)	200 (74.3%)	69 (25.7%)	229 (85.1%)	40 (14.9%)
6–7 h	100 (23.6%)	36 (36.0%)	64 (64.0%)	54 (54.0%)	46 (46.0%)	78 (78.0%)	22 (22.0%)	77 (77.0%)	23 (23.0%)	73 (73.0%)	27 (27.0%)	54 (54.0%)	46 (46.0%)	75 (75.0%)	25 (25.0%)
<6 h	55 (13.0%)	5 (9.1%)	50 (90.9%)	21 (38.2%)	34 (61.8%)	36 (65.5%)	19 (34.5%)	31 (56.4%)	24 (43.6%)	31 (56.4%)	24 (43.6%)	26 (47.3%)	29 (52.7%)	43 (78.2%)	12 (21.8%)
Habitual sleep efficiency			*p* < 0.001 *^a^*		*p* < 0.001 *^a^*		*p* < 0.001 *^a^*		*p* = 0.024 *^a^*		*p* < 0.001 *^a^*		*p* < 0.001 *^a^*		*p* < 0.001 *^a^*
≥75%	231 (54.5%)	150 (64.9%)	81 (35.1%)	171 (74.0%)	60 (26.0%)	205 (88.7%)	26 (11.3%)	206 (89.2%)	25 (10.8%)	201 (87.0%)	30 (13.0%)	174 (75.3%)	57 (24.7%)	202 (87.4%)	29 (12.6%)
65–74%	122 (28.8%)	43 (35.2%)	79 (64.8%)	69 (56.6%)	53 (43.4%)	94 (77.0%)	28 (23.0%)	99 (81.1%)	23 (18.9%)	86 (70.5%)	36 (29.5%)	70 (57.4%)	52 (42.6%)	100 (82.0%)	22 (18.0%)
<65%	71 (16.7%)	19 (26.8%)	52 (73.2%)	34 (47.9%)	37 (52.1%)	51 (71.8%)	20 (28.2%)	57 (80.3%)	14 (19.7%)	48 (67.6%)	23 (32.4%)	36 (50.7%)	35 (49.3%)	45 (63.4%)	26 (36.6%)

Notes: *^a^ p-value for trend*; PSQI: Pittsburgh Sleep Quality Index; HT: Hypertension; DM: Diabetes mellitus; NCDs: Non-communicable diseases; FBS: Fasting blood sugar; SBP: Systolic blood pressure; DBP: Diastolic blood pressure.

**Table 4:** Associations between overall sleep quality (continuous PSQI score), sleep quality components, and health-related clinical outcomes among older adults (*n* = 424).

Outcome	Variable	Univariable Analysis		Multivariable Analysis ^a^	
		COR (95%CI)	*p*-Value	AOR (95%CI)	*p*-Value
All NCDs	Overall PSQI (Points)	2.22 (1.92, 2.56)	<0.001	2.37 (2.00, 2.81)	<0.001
	Subjective sleep quality				
	Very good	1 (ref)		1 (ref)	
	Fairly bad	14.76 (8.55, 25.47)	<0.001	15.73 (8.33, 29.69)	<0.001
	Very bad	30.85 (15.60, 60.98)	<0.001	44.08 (19.50, 99.61)	<0.001
	Sleep duration				
	>7 h	1 (ref)		1 (ref)	
	6–7 h	3.10 (1.92, 5.00)	<0.001	4.07 (2.30, 7.20)	<0.001
	<6 h	17.45 (6.73, 45.22)	<0.001	16.55 (6.02, 45.52)	<0.001
	Habitual sleep efficiency				
	≥75%	1 (ref)		1 (ref)	
	65–74%	3.40 (2.15, 5.39)	<0.001	3.31 (1.98, 5.54)	<0.001
	<65%	5.07 (2.81, 9.15)	<0.001	5.40 (2.75, 10.63)	<0.001
Hypertension	Overall PSQI (Points)	1.47 (1.36, 1.60)	<0.001	1.51 (1.37, 1.66)	<0.001
	Subjective sleep quality				
	Very good	1 (ref)		1 (ref)	
	Fairly bad	7.53 (4.30, 13.20)	<0.001	7.02 (3.79, 12.99)	<0.001
	Very bad	12.74 (6.91, 23.49)	<0.001	13.53 (6.87, 26.64)	<0.001
	Sleep duration				
	>7 h	1 (ref)		1 (ref)	
	6–7 h	2.42 (1.50, 3.91)	<0.001	3.10 (1.79, 5.37)	<0.001
	<6 h	4.60 (2.51, 8.46)	<0.001	4.25 (2.19, 8.27)	<0.001
	Habitual sleep efficiency				
	≥75%	1 (ref)		1 (ref)	
	65–74%	2.19 (1.38, 3.48)	<0.001	1.97 (1.20, 3.24)	0.008
	<65%	3.10 (1.79, 5.38)	<0.001	2.73 (1.49, 5.01)	0.001
Diabetes Mellitus	Overall PSQI (Points)	1.41 (1.28, 1.54)	<0.001	1.42 (1.28, 1.58)	<0.001
	Subjective sleep quality				
	Very good	1 (ref)		1 (ref)	
	Fairly bad	7.35 (3.29, 16.43)	<0.001	6.65 (2.87, 15.39)	<0.001
	Very bad	10.45 (4.57, 23.89)	<0.001	10.38 (4.39, 24.55)	<0.001
	Sleep duration				
	>7 h	1 (ref)		1 (ref)	
	6–7 h	2.02 (1.11, 3.67)	0.021	1.87 (0.99, 3.51)	0.051
	<6 h	3.77 (1.94, 7.34)	<0.001	3.37 (1.65, 6.87)	0.001
	Habitual sleep efficiency				
	≥75%	1 (ref)	0.001	1 (ref)	0.005
	65–74%	2.35 (1.31, 4.22)	0.004	2.21 (1.21, 4.06)	0.010
	<65%	3.09 (1.60, 5.98)	0.001	2.76 (1.39, 5.48)	0.004
Other NCDs	Overall PSQI (Points)	1.89 (1.62, 2.20)	<0.001	1.94 (1.64, 2.29)	<0.001
	Subjective sleep quality				
	Very good	1 (ref)		1 (ref)	
	Fairly bad	7.07 (3.16, 15.84)	<0.001	5.53 (2.39, 12.80)	<0.001
	Very bad	5.78 (2.44, 13.69)	<0.001	5.53 (2.25, 13.60)	<0.001
	Sleep duration				
	>7 h	1 (ref)		1 (ref)	
	6–7 h	5.06 (2.51, 10.17)	<0.001	4.34 (2.11, 8.91)	<0.001
	<6 h	13.11 (6.22, 27.62)	<0.001	11.02 (5.02, 24.21)	<0.001
	Habitual sleep efficiency				
	≥75%	1 (ref)		1 (ref)	
	65–74%	1.91 (1.04, 3.54)	0.038	1.71 (0.90, 3.24)	0.101
	<65%	2.02 (0.99, 4.15)	0.054	2.03 (0.95, 4.33)	0.068
FBS	Overall PSQI (Points)	1.47 (1.34, 1.61)	<0.001	1.47 (1.33, 1.63)	<0.001
	Subjective sleep quality				
	Very good	1 (ref)		1 (ref)	
	Fairly bad	6.15 (3.09, 12.23)	<0.001	5.13 (2.49, 10.60)	<0.001
	Very bad	8.19 (4.00, 16.76)	<0.001	7.74 (3.64, 16.47)	<0.001
	Sleep duration				
	>7 h	1 (ref)		1 (ref)	
	6–7 h	2.25 (1.28, 3.93)	0.005	2.11 (1.16, 3.83)	0.015
	<6 h	4.71 (2.50, 8.87)	<0.001	4.21 (2.11, 8.38)	<0.001
	Habitual sleep efficiency				
	≥75%	1 (ref)		1 (ref)	
	65–74%	2.81 (1.62, 4.84)	<0.001	2.66 (1.50, 4.72)	0.001
	<65%	3.21 (1.71, 6.02)	<0.001	2.84 (1.46, 5.53)	0.002
SBP	Overall PSQI (Points)	1.39 (1.29, 1.50)	<0.001	1.39 (1.28, 1.51)	<0.001
	Subjective sleep quality				
	Very good	1 (ref)		1 (ref)	
	Fairly bad	7.52 (4.25, 13.28)	<0.001	6.55 (3.54, 12.12)	<0.001
	Very bad	11.83 (6.39, 21.88)	<0.001	11.33 (5.82, 22.02)	<0.001
	Sleep duration				
	>7 h	1 (ref)		1 (ref)	
	6–7 h	2.47 (1.53, 3.99)	<0.001	2.96 (1.72, 5.09)	<0.001
	<6 h	3.23 (1.78, 5.87)	<0.001	2.71 (1.41, 5.18)	0.003
	Habitual sleep efficiency				
	≥75%	1 (ref)		1 (ref)	
	65–74%	2.27 (1.42, 3.62)	0.001	2.04 (1.24, 3.37)	0.005
	<65%	2.97 (1.71, 5.16)	<0.001	2.68 (1.46, 4.92)	0.001
DBP	Overall PSQI (Points)	1.30 (1.20, 1.42)	<0.001	1.30 (1.19, 1.43)	<0.001
	Subjective sleep quality				
	Very good	1 (ref)		1 (ref)	
	Fairly bad	6.49 (3.00, 14.04)	<0.001	5.76 (2.52, 13.16)	<0.001
	Very bad	10.14 (4.60, 22.36)	<0.001	11.07 (4.68, 26.16)	<0.001
	Sleep duration				
	>7 h	1 (ref)		1 (ref)	
	6–7 h	1.91 (1.09, 3.53)	0.025	2.13 (1.14, 3.99)	0.018
	<6 h	1.60 (0.78, 3.29)	0.204	1.35 (0.62, 2.95)	0.457
	Habitual sleep efficiency				
	≥75%	1 (ref)		1 (ref)	
	65–74%	1.53 (0.84, 2.80)	0.166	1.32 (0.70, 2.48)	0.399
	<65%	4.03 (2.17, 7.48)	<0.001	3.34 (1.71, 6.55)	<0.001

Notes: Variables: All NCDs (0 = no, 1 = yes); Hypertension (0 = no, 1 = yes); Diabetes mellitus (0 = no, 1 = yes); Other NCDs (0 = no, 1 = yes); FBS (0 = <126 mg/dL, 1 = ≥126 mg/dL); SBP (0 = <130 mmHg, 1 = ≥130 mmHg); DBP (0 = <85 mmHg, 1 = ≥85 mmHg). ^a^ Adjusted for sex, age, education, sufficient income, alcohol consumption, smoking, caffeine intake. Odds ratios for the overall PSQI score represent the increase in the odds of each outcome associated with a one-point increase in the total PSQI score. Because higher PSQI scores indicate poorer sleep quality, odds ratios greater than 1 indicate higher odds of adverse health-related outcomes with worsening sleep quality.

## Data Availability

The data presented in this study are available on request from the corresponding author. The data are not publicly available due to privacy and ethical restrictions, as it contains sensitive personal and health-related information.

## Abbreviations

The following abbreviations are used in this manuscript:

PSQI	Pittsburgh Sleep Quality Index
NCDs	Non-communicable diseases
TCM	Traditional Chinese Medicine
BP	Blood pressure
SBP	Systolic blood pressure
DBP	Diastolic blood pressure
HT	Hypertension
DM	Diabetes mellitus
FBS	Fasting blood sugar
CAD	Coronary artery disease
CKD	Chronic kidney disease
